# Characterization of the complete chloroplast genome of *Malus baccata* var. xiaojinensis

**DOI:** 10.1080/23802359.2019.1638840

**Published:** 2019-07-16

**Authors:** Yuan Ren, Ming Yan, Xueqing Zhao, Xuesen Chen, Zhaohe Yuan

**Affiliations:** a Co-Innovation Center for Sustainable Forestry in Southern China, Nanjing Forestry University, Nanjing, China;; b College of Forestry, Nanjing Forestry University, Nanjing, China;; c State Key Laboratory of Crop Biology, Shandong Agricultural University, Tai’an, Shandong, China

**Keywords:** *Malus baccata* var. xiaojinensis, complete chloroplast genome, phylogenetic analysis

## Abstract

*Malus baccata* var. xiaojinensis belonging to Rosaceae is not only an ornamental tree but also an apple genotype that is highly tolerant to Fe deficiency. In this study, we reported the complete chloroplast (cp) genome of *M. baccata* var. xiaojinensis using ILLUMINA sequencing. The whole cp genome is 160,067 bp in length, containing a pair of inverted repeats (IRs) of 26,358 bp, a large single copy (LSC) region of 88,157 bp and a small single copy (SSC) region of 19,194 bp. And, the overall GC content of the cp genome was 36.56%. A total of 110 unique genes were found in the cp genome. 17 genes were duplicated in the IRs, including six protein-coding genes, seven tRNA genes, and four rRNA genes. Fourteen genes contained one intron, whereas three genes (*rps12*, *clpP*, and *ycf3*) contained two introns. The phylogenetic analysis demonstrated a close relationship between *M. baccata* var. xiaojinensis and *Malus hupehensis*.

*Malus baccata* var. xiaojinensis is a small wild apple with elegant shapes and bright flowers and red or yellow fruits (Zhi-Qin et al. 1999) that can be used as a rootstock with resistance to iron deficiency, low temperature, and drought stress (Han et al. 1994; Liu et al. 2018). Therefore, the anti-iron deficiency characteristics will make it the most valued rootstock resource in the future. Besides, the chloroplast genome has shown great potential for solving phylogenetic problems (Hao et al. 2016) and is applicable for elucidating the taxonomic characteristics of the species and origin of the hybrid progeny (Jansen et al. 2007; Givnish et al. 2010). The comparative analysis of the cp genomes toward related species helps to understand the relationship between the important traits under the control of plastid genome (Liu et al. 2013; Yan et al. 2019). In this study, we determined the complete chloroplast genome sequence of *M. baccata* var. xiaojinensis based on next-generation sequencing data and compared it with other genus chloroplast genome sequences. The complete cp genome has been deposited in GenBank with accession number MK434915.

Total genomic DNA was extracted from young leaf tissue collected from Shandong Agricultural University using the DNeasy Plant Mini Kit (Qiagen, Venlo, the Netherlands). The voucher specimen was preserved in Shandong Agricultural University (117.112, 36.194). Paired-end Illumina genomic library was prepared and sequenced using an HiSeq 2500 platform according to the manufacturer’s instructions (Illumina, San Diego, CA). The raw paired-end reads were filtered and using fastp program (Chen et al. 2018). The high-quality reads were applied to a de novo assembly performed using GetOrganelle (Jin et al. 2018). Annotation was completed by the online program GeSeq (Tillich et al. 2017) and the result was manually adjusted where necessary using Geneious (Kearse et al. 2012).

The complete cp genome was 160,067 bp in length, containing a pair of inverted repeats (IRs) of 26,358 bp, a large single copy (LSC) region of 88,157 bp and a small single copy (SSC) region of 19,194 bp. A total of 110 unique genes were found in the cp genome. Seventeen genes were duplicated in the IRs, including six protein-coding genes, seven tRNA genes, and four rRNA genes. Among these genes, 14 genes contained one intron, whereas three genes (*rps12*, *clpP*, and *ycf3*) contained two introns. The overall GC content of the cp genome was 36.56%, which the corresponding values in LSC, SSC, and IRs regions were 34.22, 30.39 and 42.69%, respectively.

The complete cp genome sequences of twelve species were downloaded from GenBank. Multiple sequences alignment was executed using MAFFT (Katoh and Toh 2010). The maximum-likelihood (ML) phylogenetic tree was constructed using the IQ-tree with the best-fit model determined using ModelFinder (Nguyen et al. 2015; Kalyaanamoorthy et al. 2017). *Pyrus spinosa* and *Pyrus pashia* were set as outgroups. The phylogenetic tree provided valuable information for understanding the relationship within genus *Malus*. Our results (Figure 1) indicated that *M. baccata* var. xiaojinensis is closely related to *M. hupehensis*.

**Figure 1.**
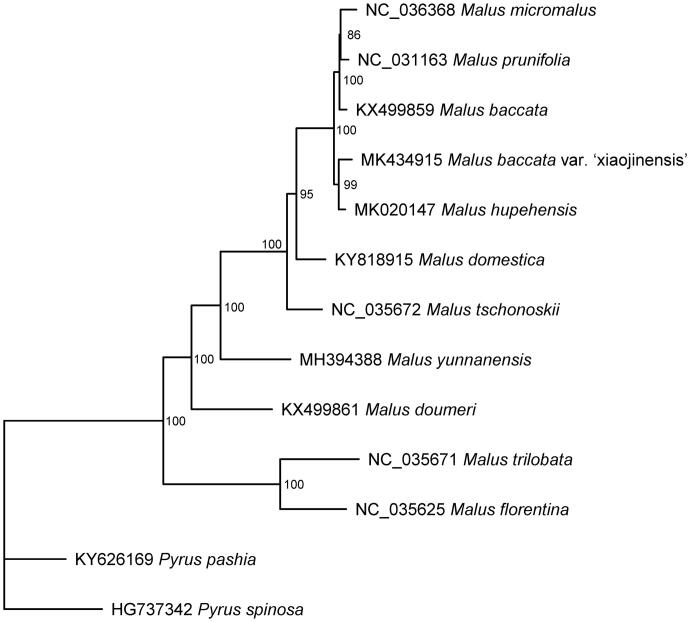
The phylogenetic tree based on the 13 complete chloroplast genomesequences.
